# Design of Polymeric Corrosion Inhibitors Based on Ionic Coumarate Groups

**DOI:** 10.1021/acsapm.0c01266

**Published:** 2021-03-19

**Authors:** Esther Udabe, Anthony Somers, Maria Forsyth, David Mecerreyes

**Affiliations:** † POLYMAT—University of the Basque Country (UPV/EHU), 20018 Donostia-San Sebastian, Spain; ‡ Institute for Frontier Materials, Deakin University, Geelong, Victoria 3220, Australia; § IKERBASQUE—Basque Foundation for Science, 48009 Bilbao, Spain

**Keywords:** corrosion inhibitors, ionic groups, polymer coatings, UV photopolymerization, functional acrylates, coumaric acid

## Abstract

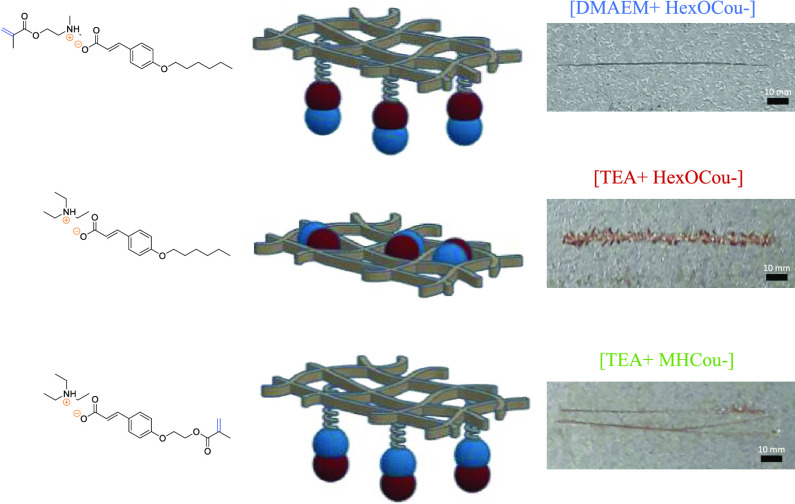

Efficient, environmentally friendly organic corrosion inhibitors are being sought to alleviate the financial loss caused by corrosion degradation of mild steel materials. Here, we show the synthesis and characterization of monomeric ionic coumarate corrosion inhibitors and their integration into polymeric acrylic UV coatings. For this purpose, we investigated the effect of including the coumarate corrosion inhibitors into the acrylic UV coating by three different means. The corrosion inhibitors could be added as a standalone ionic liquid additive, or they can be ionically attached or covalently attached to the acrylic polymer network. To achieve this, two methacrylic monomers and one nonpolymerizable ionic coumarate compound were synthesized. The anticorrosion properties of the three coumarate compounds when added to a chloride aqueous solution were investigated by various techniques. Next, the three ionic coumarate compounds were integrated into an acrylic UV polymer composition. Here, the UV coating, which shows the best anticorrosion performance, was the one where the coumarate group is attached covalently or ionically to the polymer. The UV coating, which included the coumarate compound as a nonreactive additive, presented leaching problems from the coating, limiting its anticorrosion effect. The work herein shows that the development of polymeric corrosion inhibitors that combine the barrier properties of the polymer coating and the anticorrosive identity of the organic inhibitor is a powerful strategy to prevent corrosion.

## Introduction

Corrosion is a natural process where a metal is transformed into a more stable chemical compound; however, for mild steel at least, this product is easily removed and destroys the former material.^1^ This material deterioration generates significant financial losses every year.^2^ Corrosion can be found everywhere, on food cans, pipelines, bridges, and other structures.^3^ For this reason, corrosion inhibitors, which suppress the anodic and/or cathodic electrochemical reaction, present in corrosion were developed in the last century. Chromium hexavalent is the most used inhibitor, owing to the fact that an oxide layer is created on the steel surface blocking the corrosion reaction.^4^ Despite its high anticorrosion effectiveness, hexavalent chromium is very toxic, and its use was largely prohibited, so greener alternatives are needed.^5,6^

In recent decades, new types of corrosion inhibitors have been investigated based on organic and organometallic compounds.^7,8^ Chemical structures having π electrons (such as benzene rings and double bonds) and atoms containing nonbinding electron pair (such as nitrogen, oxygen, and sulfur) are known to adsorb onto metallic surfaces by electrostatic and electronic interactions with the vacant d orbital of iron (which is present on the steel substrate).^7,8^ Due to these interactions, they can create a barrier layer on the metal, which displaces water and blocks the attack of aggressive species (e.g., Cl^–^).^9−12^ It is well known that the adsorption process of the corrosion inhibitors is enhanced by the presence of both ionic charges and long aliphatic chains.^13,14^ For this reason, tailored ionic liquids have shown an effective corrosion inhibition on mild steel surfaces. Among them, 2-methylimidazolinium *p*-coumarate presented the highest anticorrosive profile, showing a strong anodic inhibition effect.^15−19^ For this reason, the organic *p*-coumarate anion coupled with different metallic and organic cations has received considerable attention as corrosion inhibitors.^2,12,19^

On the other hand, polymer coatings are known to protect metallic surfaces from corrosion by isolating the metal from the corrosive environment. Polymer coatings based on different polymers such as polyurethanes, polyesters, or polyacrylates are habitually applied in industry.^18^ Barrier polymer coatings complement the use of corrosion inhibitors. Commonly, organic inhibitors can be introduced in a polymer coating as additives. Nevertheless, additives present some limitations such as the difficult migration or leaching issues.^19,20^ Therefore, the chemical bonding of the inhibitor into the polymer coating is an interesting methodology, which as yet has received little attention. Consequently, the chemical incorporation of corrosion inhibitors into polymer coatings is of great interest.^21,22^

Recently, a group of methacrylic ammonium coumarate molecules have shown an extraordinary anticorrosive action.^23,24^ Such monomers showed effective corrosion protection of mild steel when incorporated into a UV-curable acrylic polymer coating. The goal of this article is to investigate the different methods of attaching coumarate inhibitors into the UV polymer coatings. Initially, a nonpolymerizable ionic analogue was used as a simple additive, and we subsequently designed a monomer, which itself was a salt so that in the polymerized state, the coumarate group interacts ionically with the cationic polymer backbone. Finally, we were interested to understand the effect of covalently attaching the coumarate group to the polymer backbone and so we designed a monomer salt with the polymerizable moiety on the anion. Our final aim is to design an efficient anticorrosion system, which combines the protective properties of the polymeric coating and the anticorrosive ability of the organic inhibitor.

## Experimental Section

### Materials and Methods

#### Reagents

*p*-Coumaric acid, 2-(dimethylamino)ethyl methacrylate, potassium hydroxide, potassium iodide, 2-bromoethyl methacrylate, triethylamine, and Darocur (Speedcure 73) were obtained from Sigma-Aldrich. 1-Bromohexane was obtained from Acros Organics. Oxybis(propane-1,2-diyl) diacrylate, dipropylene glycol diacrylate, trimethylpropyl triacrylate, cyclic trimethylolpropane formal acrylate, and acid-based adhesion promoters were obtained from Arkema/Sartomer. Mild steel AS1020, NaCl aqueous solution, concentrated HCl, Milli-Q water, methanol, and ethanol were used without further purification.

#### Characterization Methods

Nuclear magnetic resonance (NMR) spectra were carried out on a Bruker AC-400 spectrometer. Attenuated total reflection-Fourier transform infrared (ATR-FTIR) spectra were recorded on Bruker Alpha-P equipment. A BioLogic VMP3 multichannel potentiostat and EC Lab V10.44 software were used for potentiodynamic polarization (PP) experiments. Potentiodynamic polymerization, open-circuit voltage (OCV), inhibitor efficiency (IE), and impedance spectroscopy were monitored using experimental details as described before.^23^ A Leica MZ 7 optical microscope and LAS V4.0 software were used to observe surfaces after 24 h of immersion. Scanning electron microscopy (SEM) and energy dispersive X-ray spectroscopy (EDS) were used to observe mild steel surfaces after the immersion test. A JSM-IT300 LV SEM instrument with attached Oxford instrument X-Max 50 mm^2^ EDS detector was used at the accelerating voltage of 20 kV. EDS spectra collected for 60 s were processed using AZtec software.

### Synthetic Methods

#### Synthesis of 2-(Dimethylamino)ethyl methacrylate *p*-hexoxy coumarate

Equimolar amounts of 2-(dimethylamino)ethyl methacrylate and 4-hexyloxycinnamic acid were weighed and mixed. The product was obtained instantly as a viscous liquid. ^1^H NMR (400 MHz, D_2_O) δ 7.55 (d, 2H, *J* = 4.0 Hz), 7.35 (d, 1H, *J* = 16.0 Hz), 6.99 (d, 2H, *J* = 8.0 Hz), 6.36 (d, 1H, *J* = 16.0 Hz), 4.08 (m, 2H), 1.74 (m, 2H), 1.32 (m, 6H), 0.85 (m, 3H).

#### Synthesis of Triethylammonium *p*-hexoxy coumarate

Equimolar amounts of triethylamine and p-hexoxy coumaric acid were weighted and mixed. The product was obtained instantly as a viscous liquid. ^1^H NMR (400 MHz, D_2_O) δ 7.70 (d, 1H, *J* = 16.0 Hz), 7.60 (d, 2H, *J* = 8.0 Hz), 7.02 (d, 2H, *J* = 8.0 Hz), 6.48 (d, 1H, *J* = 8.0 Hz), 6.28 (s, 1H), 5.72 (s, 1H), 4.49 (t, 2H, *J* = 4.0 Hz), 4.10 (t, 2H, *J* = 4.0 Hz), 2.98 (t, 2H, *J* = 4.0 Hz), 2.58, (s, 6H), 2.08 (s, 3H), 1.91 (m, 2H), 1.48 (m, 6H), 1.06 (m, 3H).

#### Synthesis of Triethylammonium p-4-ethyloxymethacrylate coumarate

*p*-Coumaric acid (1 mol), KOH (3 mol), and a catalytic amount of KI were dissolved in a mixture of ethanol/water (75/25%) and refluxed for 1 h. 2-Bromoethyl methacrylate (1 mol) was added, and the reaction mixture was refluxed for a further 24 h. The solvent was removed, and the precipitate was acidified with concentrated HCl. The crude product was filtered, washed with water, and recrystallized from a mixture of ethanol/water (75/25%). The product, p-ethyloxymethacrylate coumaric acid, was dried under vacuum and obtained as a white powder.

Equimolar amounts of triethylamine and *p*-ethyloxymethacrylate coumaric acid were weighed and mixed. The product was obtained instantly as a viscous liquid. ^1^H NMR (400 MHz, D_2_O) δ 7.56 (d, 2H, *J* = 8.0 Hz), 7.34 (d, 1H, *J* = 16.0 Hz), 7.01 (d, 2H, *J* = 8.0 Hz), 6.39 (d, 1H, *J* = 16.0 Hz), 4.15 (t, 2H, *J* = 4.0 Hz), 3.90 (t, 2H, *J* = 4.0 Hz), 2.68 (q, 6H, *J* = 8.0 Hz), 1.88 (s, 3H), 1.04 (t, 9H, *J* = 8.0 Hz).

### Preparation of Polymer Coatings by UV Photopolymerization

A typical composition includes coumarate monomer (20 wt %) and an acrylic monomer composition described before (80 wt %) in the presence of a Darocur^23^ (Speedcure 73) photoinitiator.

Acetone was used to degrease AS1020 mild steel substrates. The aforementioned monomer solution was cast onto a metallic plate and UV-cured for 120 s using a UVC-5 (DYMAX) UV Curing Conveyor System with an intensity of up to 400 mW/cm^2^, a lamp-to-belt distance of 30 mm, and a belt speed of 7 m/min.

## Results and Discussion

### Ionic Coumarate Compounds as Corrosion Inhibitors

#### Potentiodynamic Polarization (PP)

**Figure 1:**
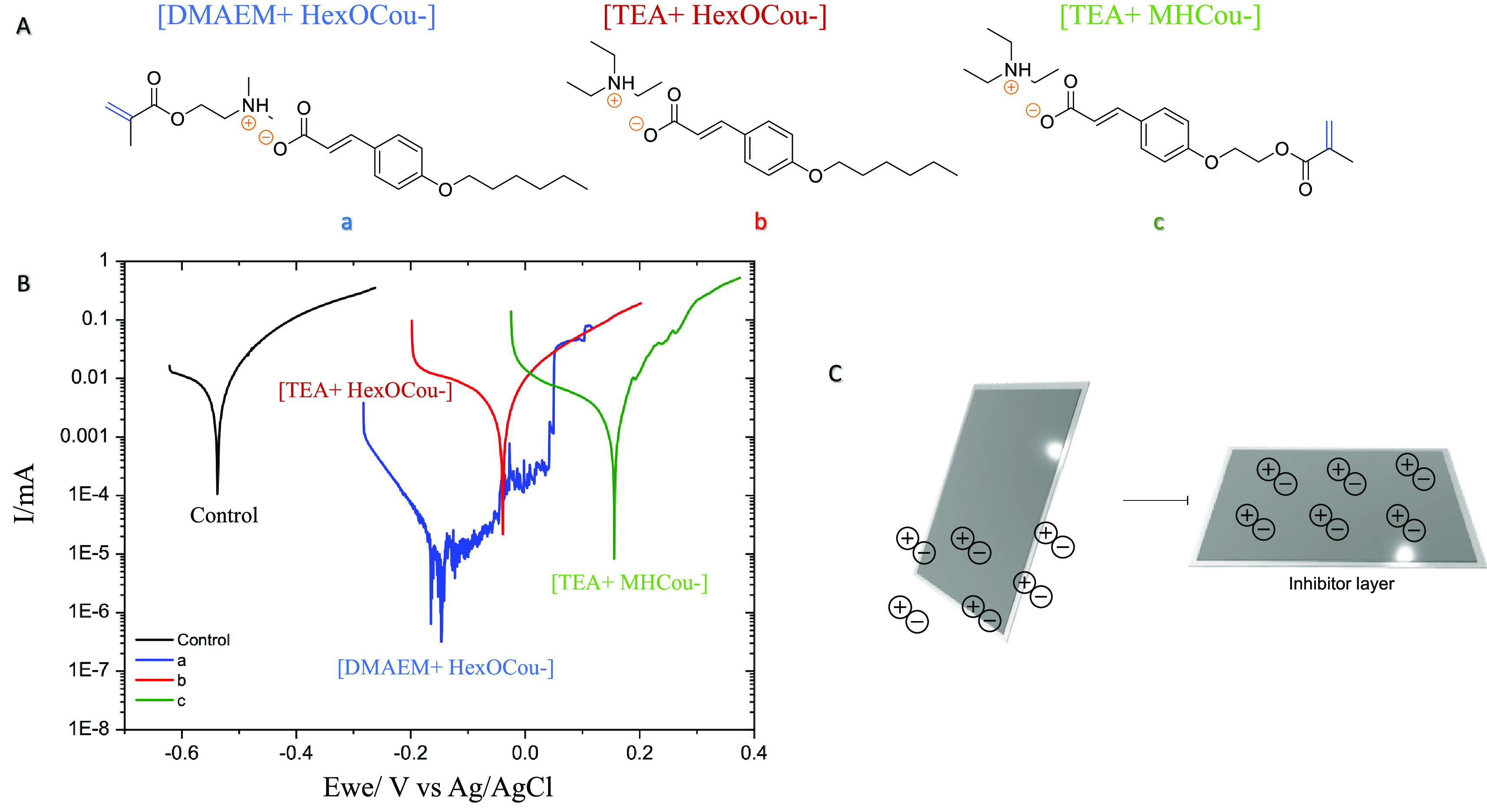
(A) Chemical structures of monomers. (B) Potentiodynamic polarization results of AS1020 mild steel after 24 h at OCV in control (black) and inhibited solutions containing 8 mM [DMAEM+HexCou−] (a: blue), [TEA+HexCou−] (b: red), and [TEA+MHCou−] (c: green). (C) Graphical representation of mild steel immersed in an aqueous solution containing ionic inhibitors.

**Scheme 1:**
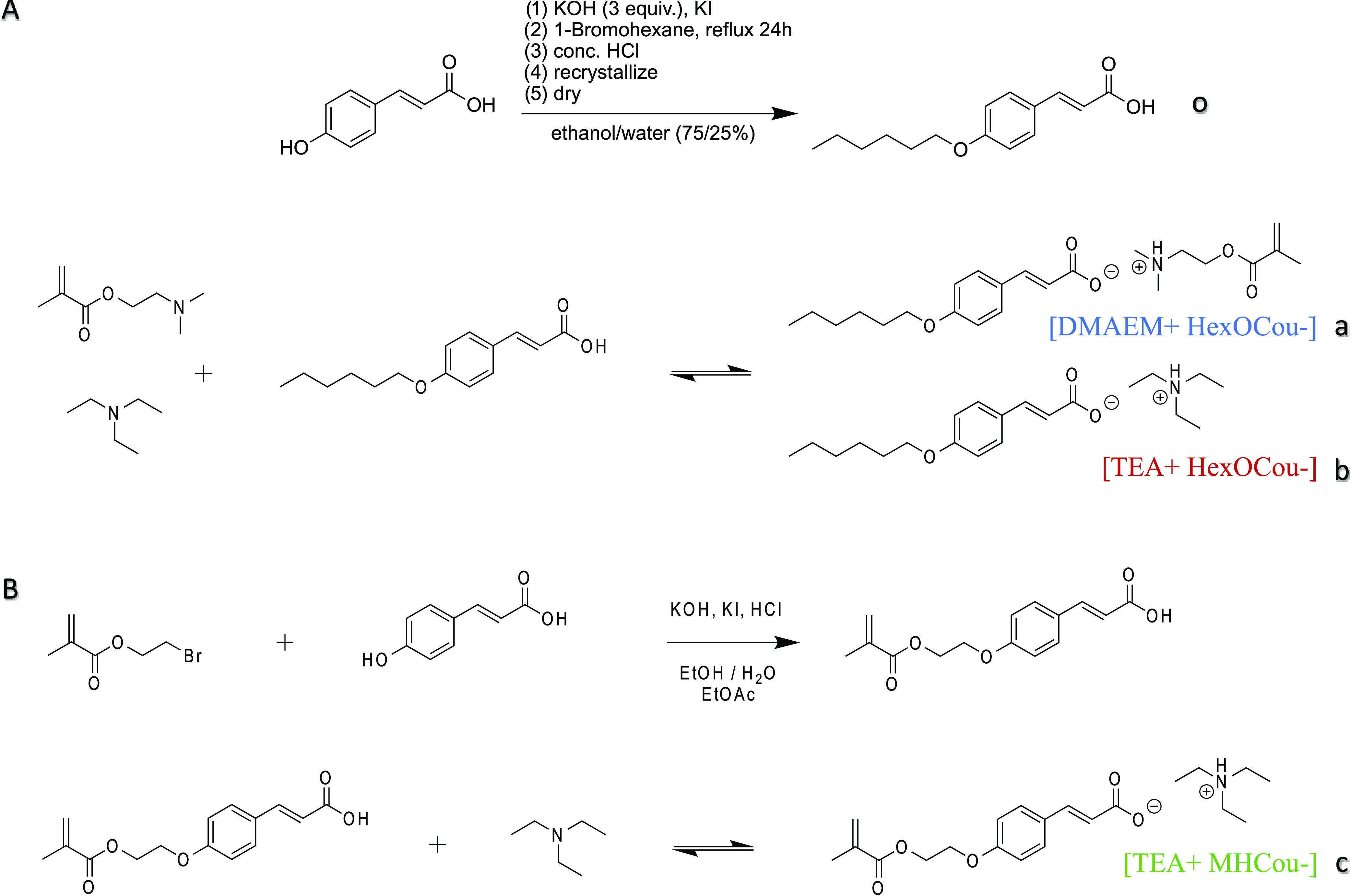
(A) Synthesis of *p*-Hexoxy Coumaric Acid (Compound o), 2-(Dimethylamino)ethyl methacrylate *p*-hexoxy coumarate (Compound a), and Triethylamonium *p*-hexoxy coumarate (Compound b) and (B) Synthesis of Triethylammonium *p*-(2-(methacryloyloxy)ethoxy) coumarate (Compound c)

As the goal of this article is to research different methods of integrating the coumarate-based inhibitor in a photopolymerizable polymer coating, three different *p*-coumarate anion-based inhibitors were synthesized as shown in Scheme 1. First, [DMAEM+HexCou−] was synthesized as an inhibitor, which incorporates the *p*-coumarate through ionic interaction with the cationic polymer. Second, [TEA+HexCou−] was designed as a nonpolymerizable inhibitor additive. Finally, [TEA+MHCou−] was synthesized as a potential inhibitor in which the coumarate group is covalently attached to the acrylic polymer backbone. The chemical structures are also shown in Figure 1A. The properties of these compounds as corrosion inhibitors in solution were investigated in the first instance. Figure 1 shows a comparison of the PP data for AS1020 mild steel electrodes after 24 h exposure in control solution (0.01 M NaCl aqueous solution without inhibitor) and inhibited solution (0.01 M NaCl + 8 mM inhibitor monomers).

These three new ionic inhibitors were tested in solution to analyze the anticorrosive properties of the created inhibitor layer on the metallic surface (Figure 1C). As it can be observed, these compounds mainly suppress the anodic reaction of the corrosion due to the shift of the corrosion potential (*E*_corr_) toward higher potential values compared with the control. The control sample presents *E*_corr_ at −523 mV (vs Ag/AgCl), and the corrosion potentials of the samples containing inhibitors are −146 mV ([DMAEM+HexCou−]), −39 mV ([TEA+HexCou−]), and 155 mV ([TEA+MHCou−]). The corrosion current *i*_corr_ was shifted to lower values, presenting positive inhibitor efficiencies. As presented in our previous work,^24^ the cationic component of these salts has a great impact on the anticorrosive profile, due to the different interaction that they have with the metallic substrate. In this work, it can also be observed that changing the cationic part changes the inhibitor efficiency. For instance, [DMAEM+HexCou−] presents an efficiency of 99.1%, whereas the other inhibitors present efficiencies of 59.5 and 69.2% for [TEA+HexCou−] and [TEA+MHCou−], respectively.

#### Electrochemical Impedance Spectroscopy (EIS) Measurements

The anticorrosive ability of the different compounds was more deeply investigated by EIS tests. The impedance of samples was measured during immersion in 0.01 M NaCl aqueous solution for 24 h. As observed in the PP plots, Nyquist plot (Figure S1) shows that [DMAEM+HexCou−] presents the largest semicircle. Further, the impedance remains constant after 24 h immersion. On the other hand, [TEA+HexCou−] and [TEA+MHCou−] inhibitors do not have the same anticorrosive performance as [DMAEM+HexCou−], as they present after 24 h of immersion a similar impedance response to the control sample. As mentioned before, and as is consistent with the PP plot, the interaction between the cation, anion, and substrate is crucial in anticorrosion terms, so changing the nature of the molecules in the inhibitor modifies the inhibition efficiency.

**Figure 2:**
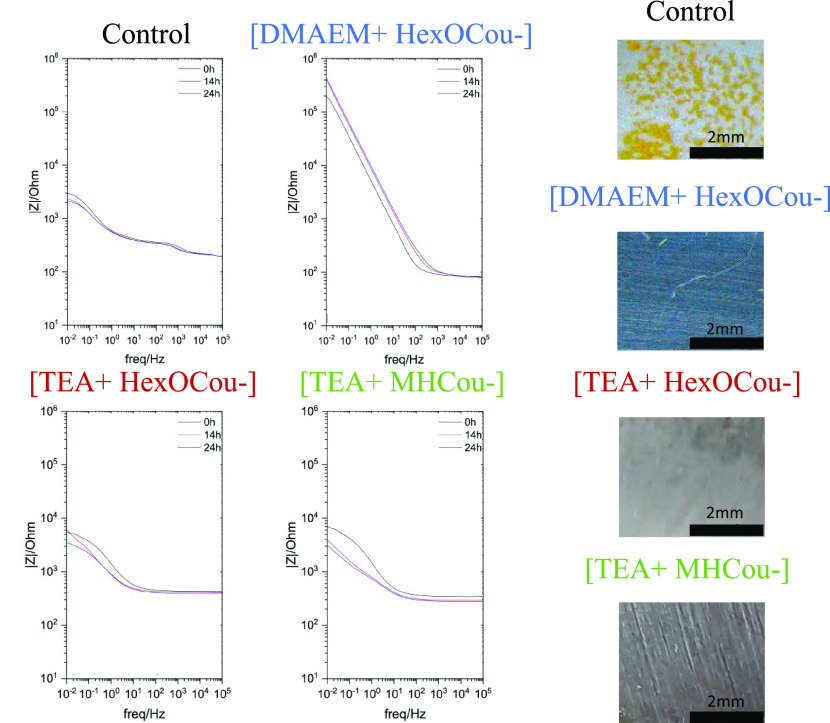
Electrochemical impedance spectra (impedance modulus plots) and optical microscopy images for AS1020 mild steel immersed in the control and inhibited solutions up to 24 h.

The Bode impedance plots of the control and different inhibitors are shown in Figure 2, along with optical images of the surfaces following 24 h immersion. In the low-frequency range for the control sample, the impedance and phase angle plateau (Figure S2) decrease, respectively, from 10^3.48^ Ω cm^2^ and 35° at 2 h immersion to 10^3.34^ Ω cm^2^ and 30° at 24 h immersion. Metallic substrates immersed in inhibitor solutions present different anticorrosive responses due to variation in the inhibitor film formed on the metallic surface. As observed before, the [DMAEM+HexCou−] ionic liquid showed the highest impedance value in the low-frequency range at 24 h immersion, 10^5.64^ Ω cm^2^. In the phase angle (Figure S1), a plateau is observed at 74°, which demonstrates high capacitive behavior and anticorrosive performance.

Mild steel, after being immersed for 24 h in [TEA+HexCou−] solution, shows an impedance of 10^3.54^ Ω cm^2^ and a corresponding phase angle of 33°. The Bode plot regarding [TEA+MHCou−] monomer shows an impedance of 10^3.41^ Ω cm^2^ that corresponds to a phase angle of 32° after 24 h immersion. The nonpolymerizable inhibitor additive [TEA+HexCou−] and the inhibitor that can be covalently attached to the polymer backbone [TEA+MHCou−] are showing a similar response to the control; thus, the blocking effect to the media that these two inhibitors are giving the surface is the same as the control.

#### Immersion Tests

##### Optical Microscopy, SEM, and EDS Analyses

**Figure 3:**
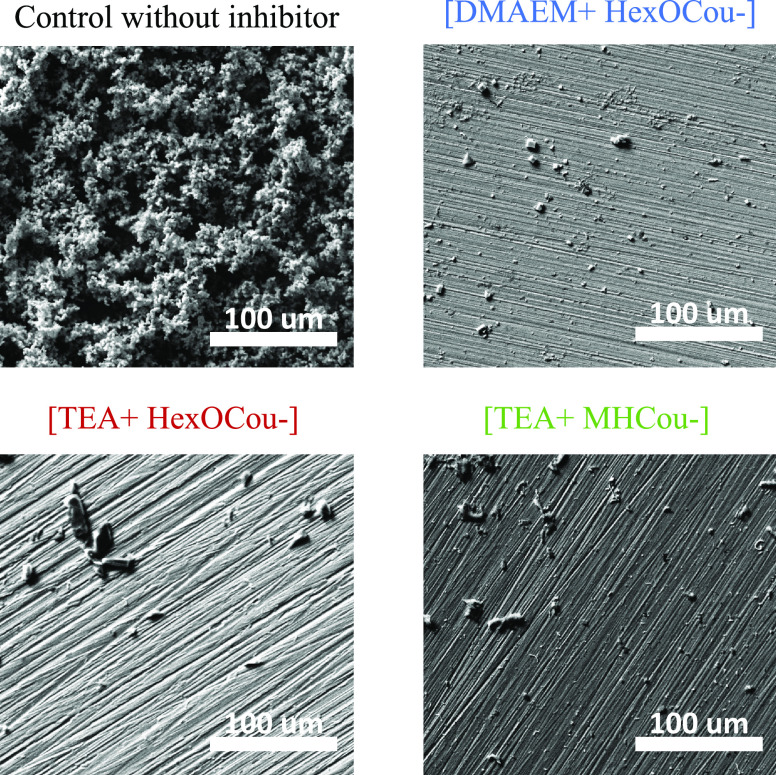
SEM images of AS1020 mild steel after an exposure of 24 h in 0.01 M NaCl control solution and inhibitors containing solutions (0.01 M NaCl + 8 mM inhibitor monomers).

Mild steel AS1020 surfaces were studied after an immersion of 24 h with and without inhibitors by optical microscopy and scanning electron microscopy. In Figures 2 and 3, optical and SEM images of metal immersed in control solution shows rust deposits. The EDS data, Figure S3, corroborate that those precipitates are mainly iron oxide. On the other hand, the surfaces in contact with solutions containing inhibitors show less corrosion products, and the EDS data confirm that carbon, oxygen, and nitrogen atoms are present in the deposits observed in SEM images. Thus, this indicates that an inhibitor interaction layer is created on the metallic surface.

### Acrylic UV Polymer Coatings Including the Coumarate Ionic Compounds

**Figure 4:**
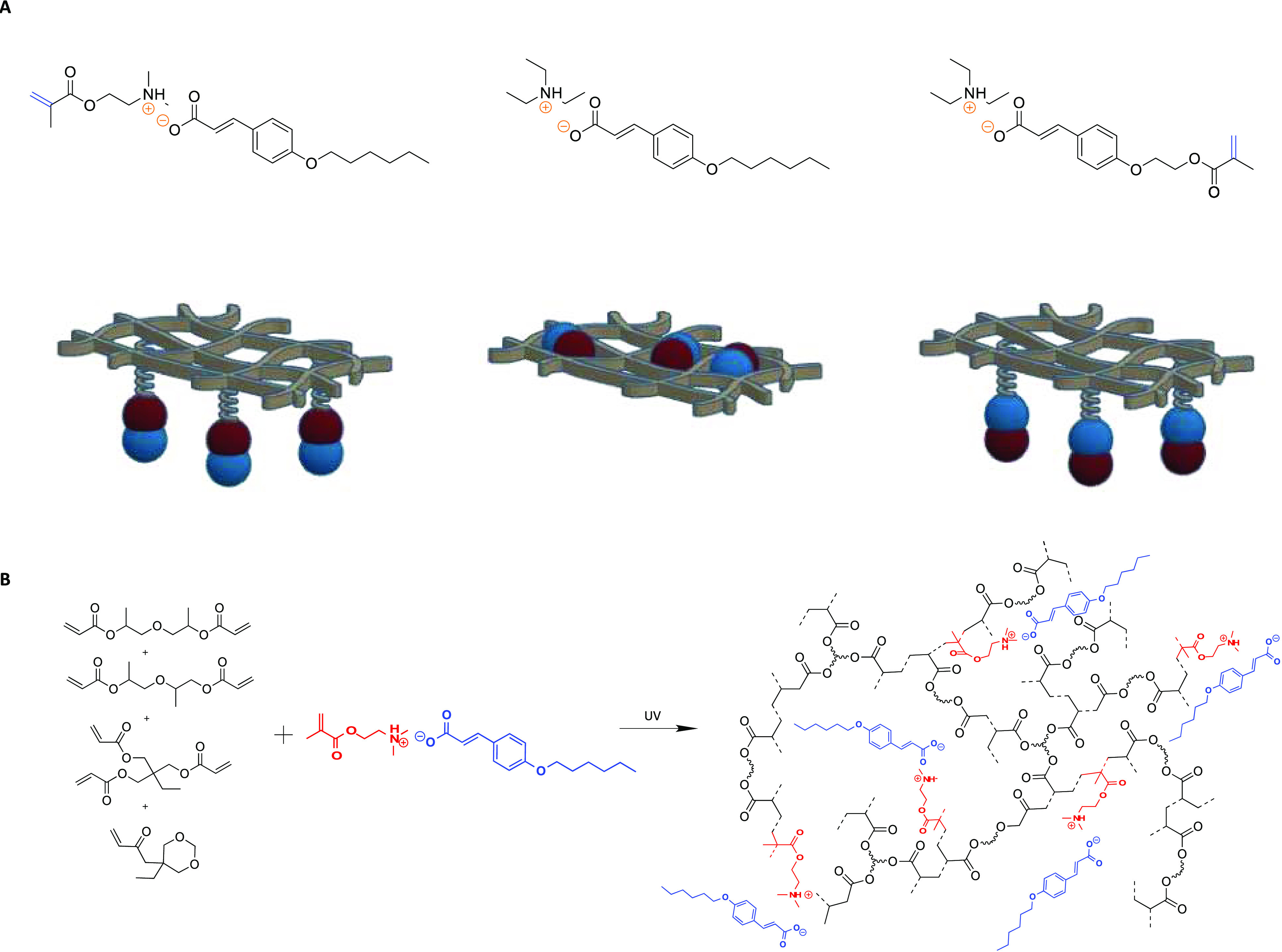
(A) Monomer and polymer representations. (B) Photopolymerization example of representative UV polymer coating.

The three different coumarate-based inhibitors were incorporated by photopolymerization (Figure 4A) into a typical UV-curable acrylic formulation, which consists of a mixture of mono-, di-, and trifunctional acrylic monomer and a photoinitiator (Figure 4B). Polymer coatings were formed onto the mild steel AS1020 surface by photopolymerization. After UV-curing the monomer liquid mixture, transparent acrylic coatings were obtained onto the metal surface. The acrylic double-bond polymerization was confirmed by ATR-FTIR spectra. The ATR-FTIR spectra of all coatings containing the control coating, the monomer mixture, and the polymer coating can be seen in Figure S4. High-yield photopolymerization (>90%) was confirmed due to the disappearance of the band between 1600 and 1650 cm^–1^ associated with the acrylic double bond. By this method, polymer coatings containing 20 wt % of the ionic coumarate inhibitors were easily obtained. First, in the case of [DMAEM+HexCou−], the cationic parts polymerized and the coumarate had an ionic interaction with the polymer. Second, in the case of [TEA+HexCou−], the nonpolymerizable inhibitor was added just as an additive to the acrylic network. Third, in the case of [TEA+MHCou−], the anionic coumarate group is covalently attached to the acrylic network.

#### Stability of UV Polymer Coatings in Water

The acrylic polymer coatings containing the different ionic additives were immersed in water for 24, 48, and 72 h to investigate their stability. From this simple test, water uptake or leaching of the ionic coumarate compounds from the acrylic coating can be studied. The weight of the control coating without ionic additives and 20 wt % containing [DMAEM+HexCou−] after immersion for 72 h in 1 M NaCl remains constant, meaning that neither swelling nor leaching occurred. On the other hand, for the [TEA+HexCou−] 20 wt %-containing coating, a weight loss of 22% can be observed after 72 h of immersion. This can be attributed to the complete leaching of the additive, which anticipates the poor anticorrosive activity. The coating containing 20 wt % [TEA+MHCou−] showed a weight loss of 11%, which can be attributed to the loss of some unreacted monomer or the bulky TEA+ cation.

#### Scribe Test

**Figure 5:**
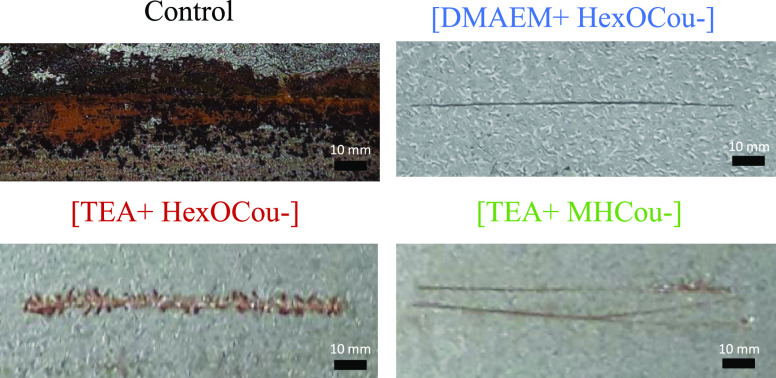
Filiform test images of UV polymer coatings including 20 wt % of the different ionic coumarate compounds.

**Figure 6:**
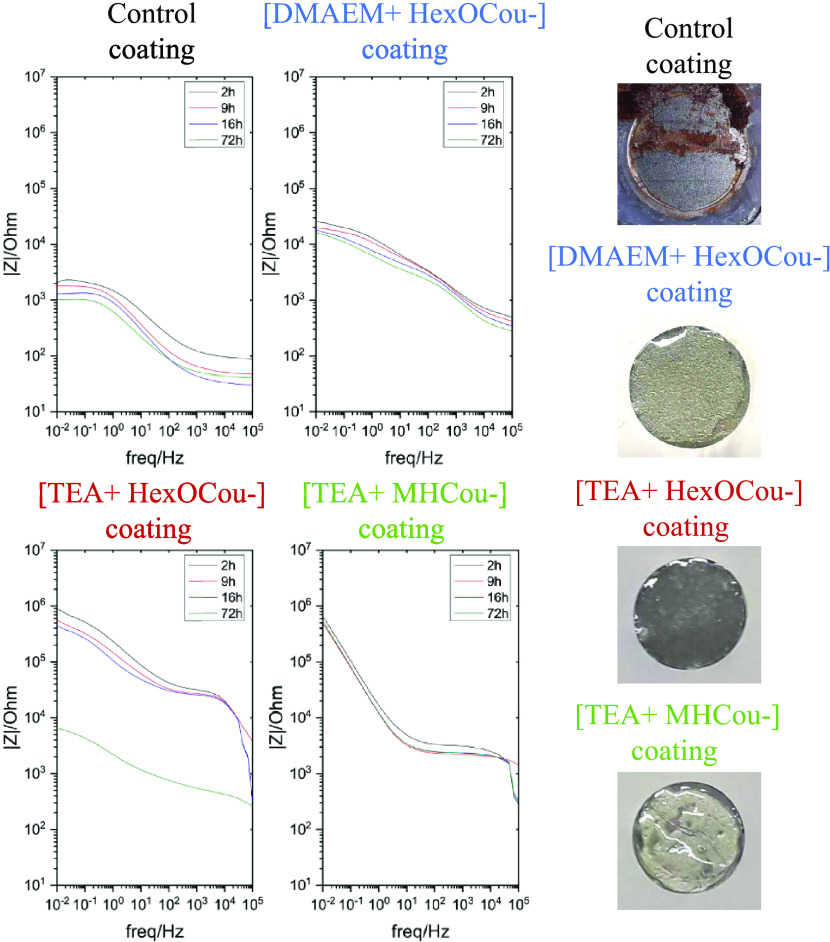
(Left) Electrochemical impedance spectra for different polymer coatings on AS1020 mild steel immersed in 0.005 M NaCl: impedance modulus plots for inhibited coating without inhibitors (control) and containing 20% [DMAEM+HexCou−], 20% [TEA+HexCou−], and 20% [TEA+MHCou−] immersed in 0.005 M NaCl after 72 h. (Right) Optical images of polymer coatings on AS1020 mild steel without inhibitors (control) and containing 20% [DMAEM+HexCou−], 20% [TEA+HexCou−], and 20% [TEA+MHCou−] immersed in 0.005 M NaCl after 72 h.

The anticorrosion profile of different coatings was studied by performing a scribe test. A defect was introduced in the control coating and coatings containing 20 wt % [DMAEM+HexCou−], [TEA+HexCou−], and [TEA+MHCou−], respectively. After 10 days of exposure to 85% humidity following acid activation, images of all coating were taken (Figure 5). The control coating, which does not present any inhibitor in its formulation, showed a fully rusted surface. In contrast, the polymer coatings containing inhibitors presented with little corrosion propagation and with almost negligible corrosive defects. As can be observed in Figure 6, the best anticorrosive performance was obtained by coatings containing 20 wt % [DMAEM+HexCou−] and 20 wt % [TEA+MHCou−]. On the other hand, the polymer coating containing 20 wt % [TEA+HexCou−] still shows evidence of filiform corrosion. These differences can be attributed to the fact that, for coatings containing 20 wt % [DMAEM+HexCou−] and 20 wt % [TEA+MHCou−], the inhibitor is attached to the polymer ionically and covalently, thus always providing a reservoir of the inhibitor within the polymer. For the polymer containing 20 wt % [TEA+HexCou−], the inhibitor is added as an additive, which can be removed from the coating, as observed in the leaching test, hence showing poorer corrosion protection ability compared with the other two cases.

#### Electrochemical Impedance Spectroscopy

Electrochemical impedance spectroscopy (EIS) measurements were done to test the anticorrosion performance of each polymer coating. Nyquist plots (Figure S5) were obtained after immersing the polymer coatings for 72 h in 0.005 M NaCl aqueous solution.

Nyquist plots (Figure S5) and Bode plots (Figure 6) exhibit the impedance response of each polymer coating. The control coating showed a diminution in the impedance and in the plateau of the phase angle in the 10^–1^–10^1^ Hz range, which indicates the initiation of corrosion. Moreover, pictures of each polymer coating were taken after EIS measurements (Figure 6). As mentioned before, rust deposits on the surface are present on the control coating, which correlates with the data reported in the Bode plots. On the contrary, the polymer coatings containing 20 wt % [DMAEM+HexCou−], [TEA+HexCou−], and [TEA+MHCou−] did not suffer any obvious deterioration.

From the impedance data, it appears that the best performing anticorrosive polymer coating was the one containing 20 wt % [TEA+MHCou−] inhibitor, which is attached to the polymer backbone covalently. It presents an impedance value of 10^5.72^ Ω cm^2^ after 72 h of immersion in the 10^–1^–10^1^ Hz range and a phase angle of 74°. On the other hand, 20 wt % [TEA+HexCou−] polymer coating presents an impedance value of 10^5.95^ Ω cm^2^ and a phase angle of 37° in the 10^–1^–10^1^ Hz range with 2 h of immersion in a 0.005 M NaCl aqueous solution, which are decreased to 10^3.81^ Ω cm^2^ and 27° after 72 h, respectively, which correlate with the data shown in the Nyquist plot. This inhibitor is added by simple mixing into the coating formulation, meaning that the compound is not polymerizable in the polymer matrix. As observed in the leaching test, this inhibitor can be lost after 72 h immersion, which explains the large drop in impedance seen from 16 to 72 h. The polymer coating containing 20 wt % [DMAEM+HexCou−] presents an impedance value of 10^4.2^ Ω cm^2^ in the 10^–1^–10^1^ Hz range, after 72 h of immersion in NaCl 0.005 M aqueous solution. However, in the phase angle plots (Figure S6), various peaks can be observed that can be attributed to various mechanisms involved in the anticorrosion process. In this case, the coumarate-based inhibitor is attached to the coating ionically that may show different interactions through the coating and the metal. This interaction directly affects the mechanism involved in the inhibition processes, which are not easily analyzed but can be assumed due to the appearance of different peaks in the phase angle plot.

## Conclusions

In this article, the effect of incorporating coumarate corrosion inhibitors into an acrylic UV coating was investigated. Inhibitors were added simply as an additive, ionically attached or covalently attached to the acrylic polymer coating. For this purpose, two different ionic liquid methacrylic monomers and one nonpolymerizable ionic coumarate compound were synthesized. The corrosion inhibition of the monomers in solution and in coatings on the AS1020 mild steel surface were studied by potentiodynamic polarization, electrochemical impedance spectroscopy experiments, and surface analyses. With regard to monomers, the most promising inhibitor was [DMAEM+HexCou−], which shows an inhibition efficiency of 99.1% in solution. All inhibitors were integrated in a typical acrylic coating formulation and deposited onto stainless steel by photopolymerization. The acrylic UV coatings, which showed the best anticorrosion performance, included the coumarate group attached covalently or ionically to the acrylic network. This showed that the inhibitor compound was able to protect the mild steel while being linked to the polymer. The UV coating, where the nonpolymerizable coumarate compound was added as an additive without any covalent link or strong interaction, presented leaching problems, limiting its anticorrosion effect. Overall, this article concludes that the development of polymeric corrosion inhibitors that combine the protective properties of the polymer coating and the anticorrosion effect of the organic inhibitor is a valid strategy against corrosion.

## Supporting Information Available

Electrochemical impedance spectra for AS1020 mild steel immersed in the control and inhibited solutions up to 24 h: phase angle plots, EDS data of four different zones (1: rust deposits; 2–4: inhibitor deposits), bare mild steel, and mild steel covered with each inhibitor after 24 h immersion in 0.01 M NaCl; chemical structures of a typical UV photopolymerizable acrylic formulation used in this project; and ATR-FTIR spectra and electrochemical impedance spectra for different polymer coatings: phase angle plots for inhibited coating without inhibitors (control) and containing 20% [DMAEM+HexCou−], 20% [TEA+HexCou−], and 20% [TEA+MHCou−] immersed in 0.005 M NaCl after 24 h (PDF)

## Abbreviations

[DMAEM+HexCou−]: 2-(dimethylamino)ethyl methacrylate *p*-hexoxy coumarate
[TEA+HexCou−]: triethylammonium *p*-hexoxy coumarate
[TEA+MHCou−]: triethylammonium *p*-4-ethyloxymethacrylate coumarate
PP: potentiodynamic polarization
EIS: electrochemical impedance spectroscopy
SEM: scanning electron microscopy
EDS: electron diffraction spectroscopy
