# Supplementary material for: Design of Polymeric Corrosion Inhibitors Based on Ionic Coumarate Groups
Source: ACS Appl Polym Mater. 2021 Mar 19;3(4):1739–46. doi: 10.1021/acsapm.0c01266 (PMC9164200; doi:10.1021/acsapm.0c01266)
Supplement: Supplementary file 1 — ap0c01266_si_001.pdf [file ap0c01266_si_001.pdf]

SUPPORTING INFORMATION

Design of Polymeric Corrosion Inhibitors based on Ionic Coumarate Groups

Esther Udabe [1], Anthony Somers [2], Maria Forsyth[1,2,3,] and David Mecerreyes [1,3,*]*

[1] POLYMAT University of the Basque Country UPV/EHU, Donostia-San Sebastian 20018, Spain;

[2] Institute for Frontier Materials, Deakin University, Geelong, Victoria, 3220, Australia,

[3] IKERBASQUE Basque Foundation for Science, Bilbao, Spain

***** Correspondence: (M.F.) maria.forsyth@deakin.edu.au; (D.M) David.mecerreyes@ehu.es

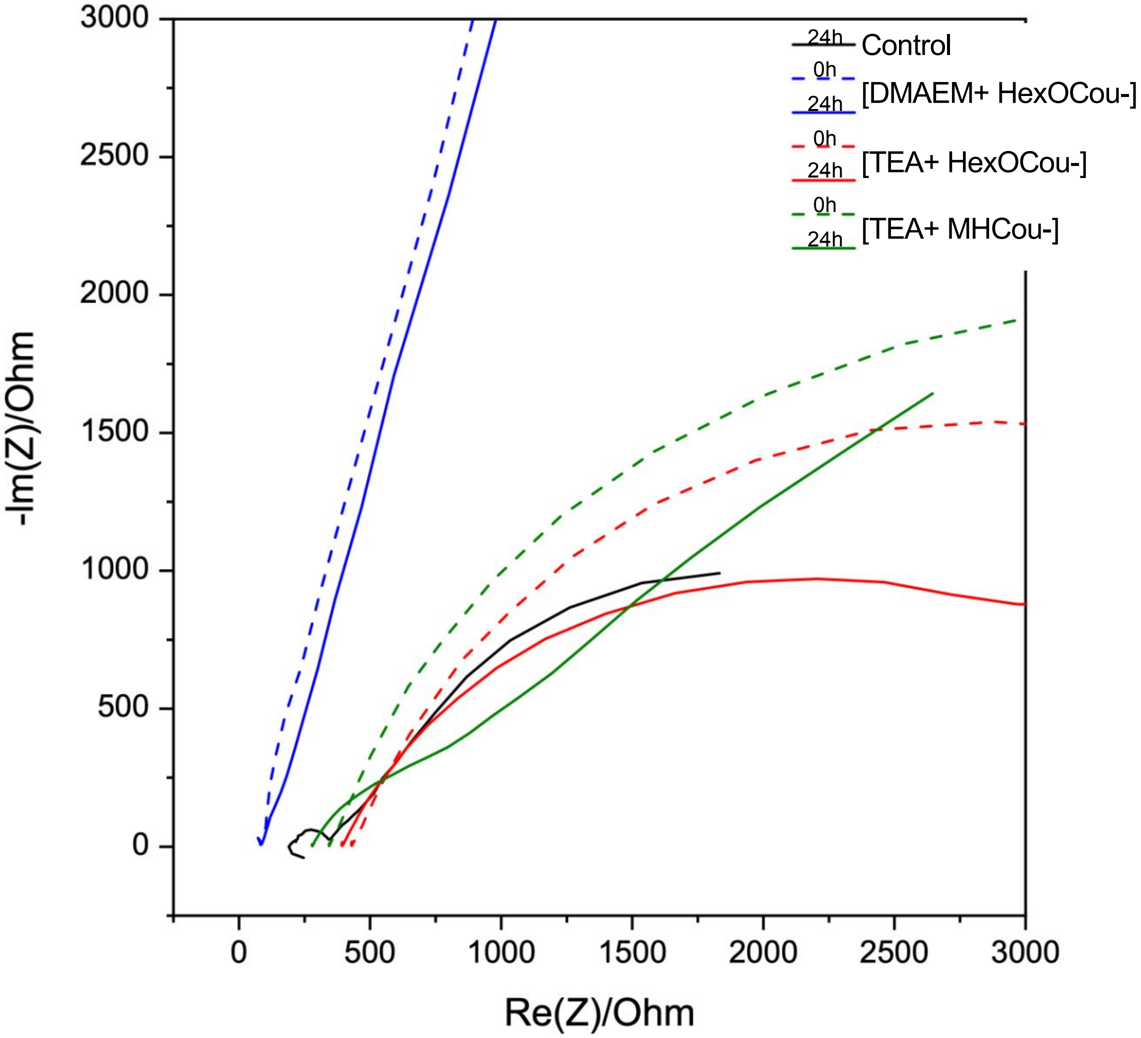

Figure S1. Electrochemical impedance spectra for AS1020 mild steel immersed in the control and the inhibited solutions up to 24 h: Nyquist plot

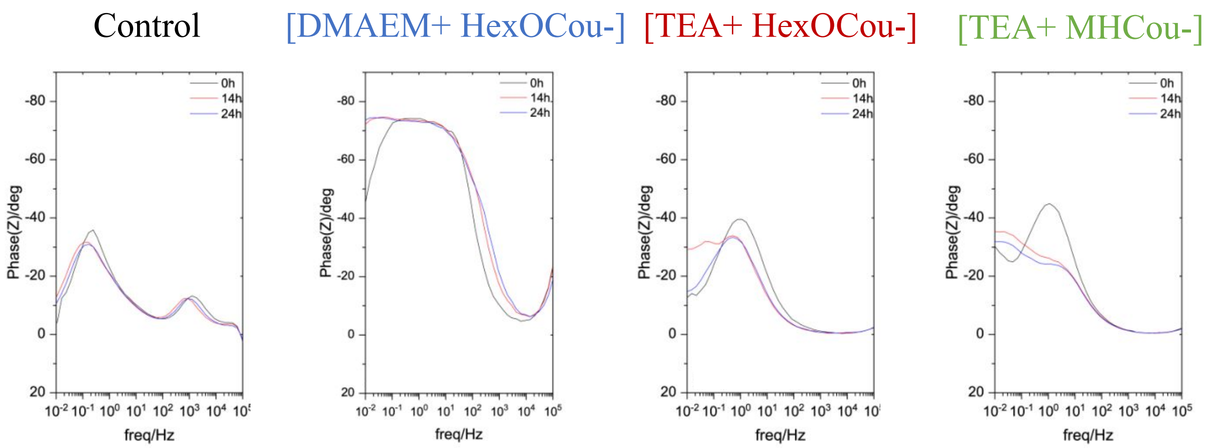

Figure S2. Electrochemical impedance spectra for AS1020 mild steel immersed in the control and the inhibited solutions up to 24 h: phase angle plots

Bare Mild Steel after 24h
immersed in 0,01 M NaCl

Control without inhibitor

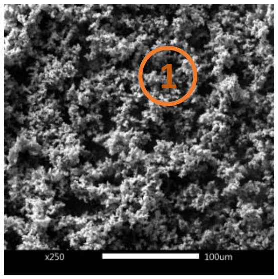

	1	2	3	**4**
	Wt %			
Fe	88,8	37,9	35,3	33,6
O	5,5	5,8	6,2	5,9
C	4,1	47,7	44,5	46,8
N	-	6,8	6,3	6,4

Mild Steel covered with inhibitor after 24h
immersed in 0,01 M NaCl

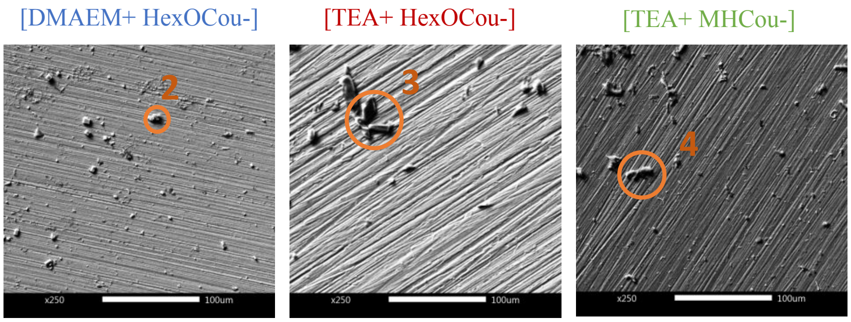

Figure S3. EDS data of 4 different zones (1: Rust deposits; 2, 3, 4: inhibitor deposits) bare mild steel and mild steel covered with each inhibitor after 24h immersed in 0,01M NaCl

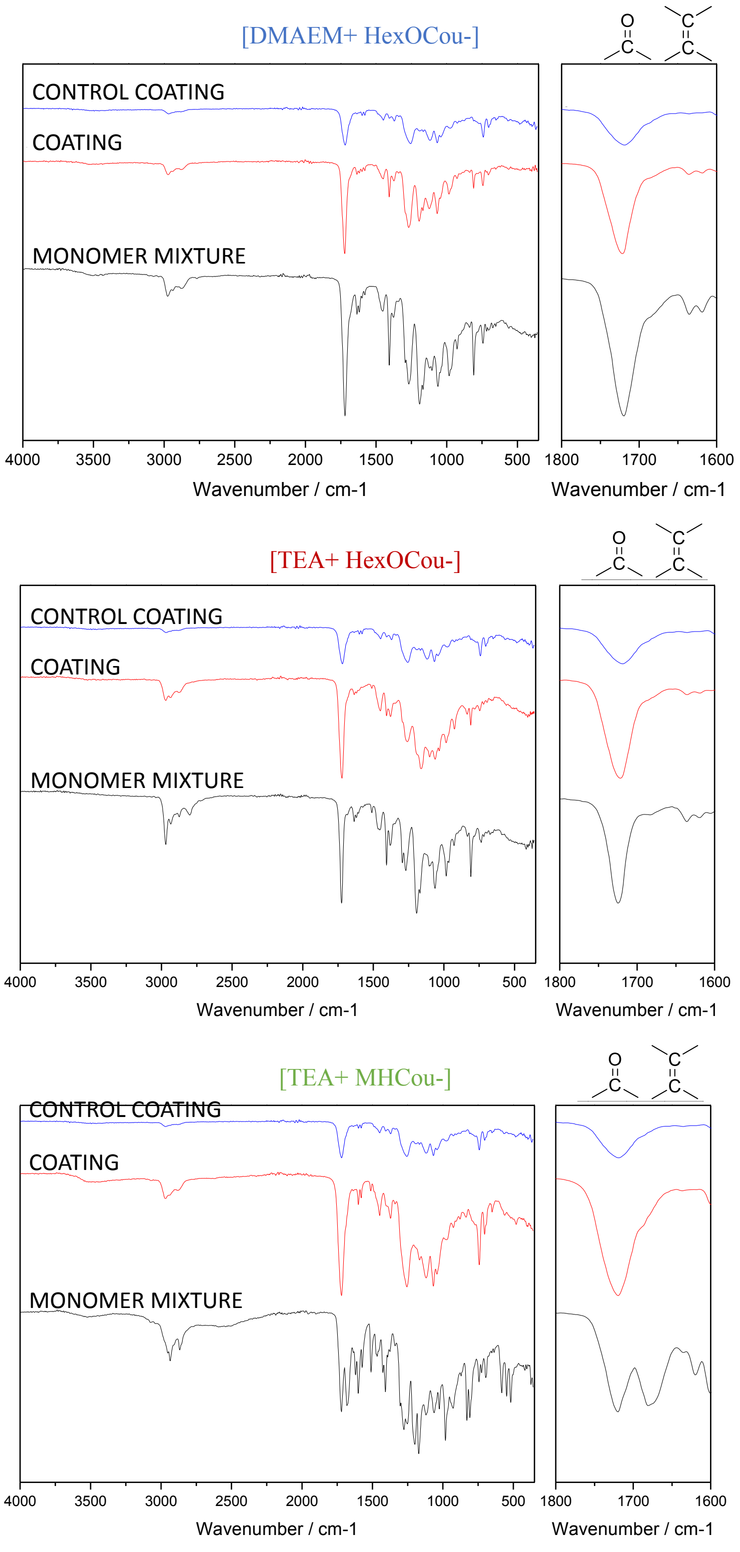

Figure S4. ATR-FTIR of inhibitors based monomer mixture and coatings compared to the control coating

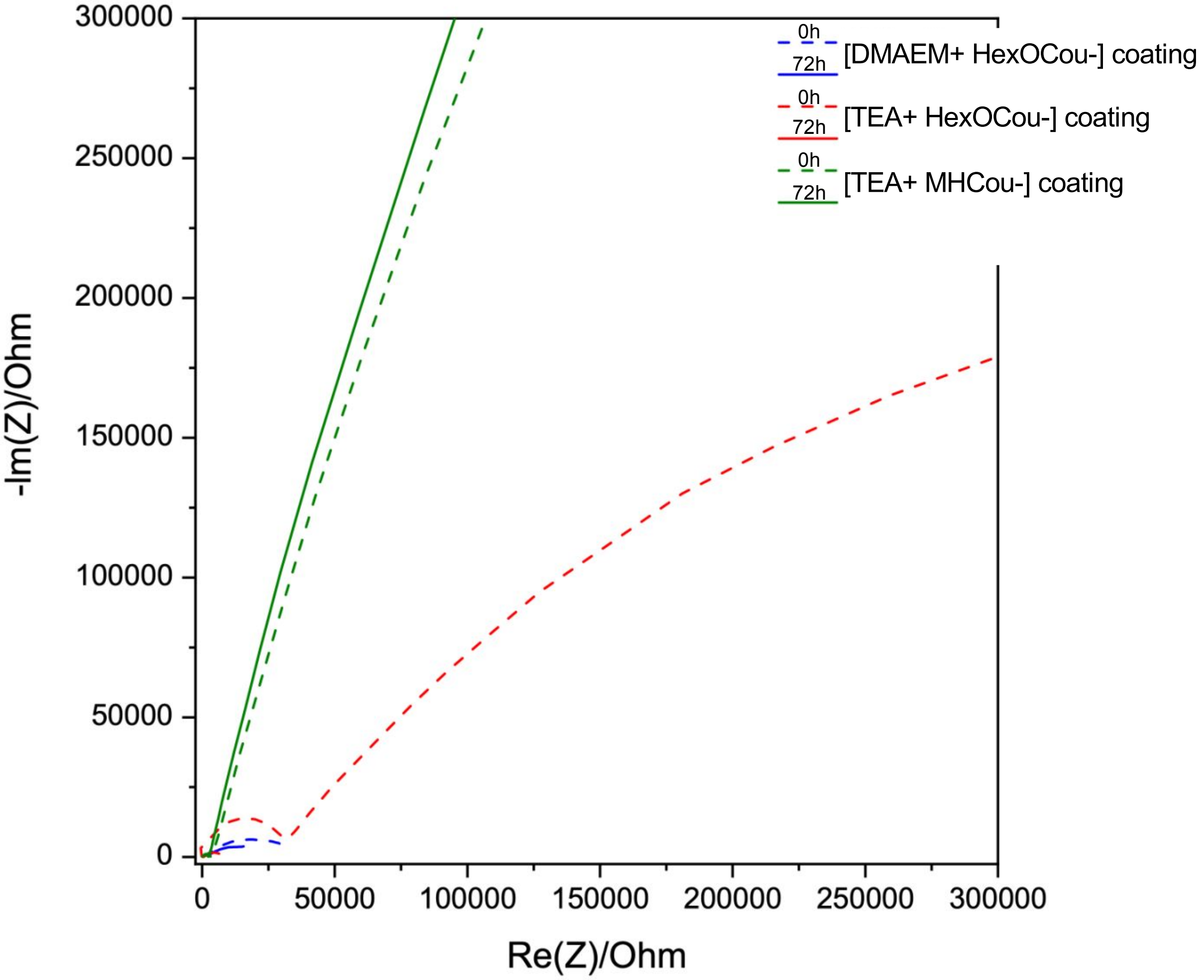

Figure S5. Nyquist plots for different polymer coatings on AS1020 mild steel immersed in 0.005M NaCl; inhibited coatings containing 20% of inhibitor.

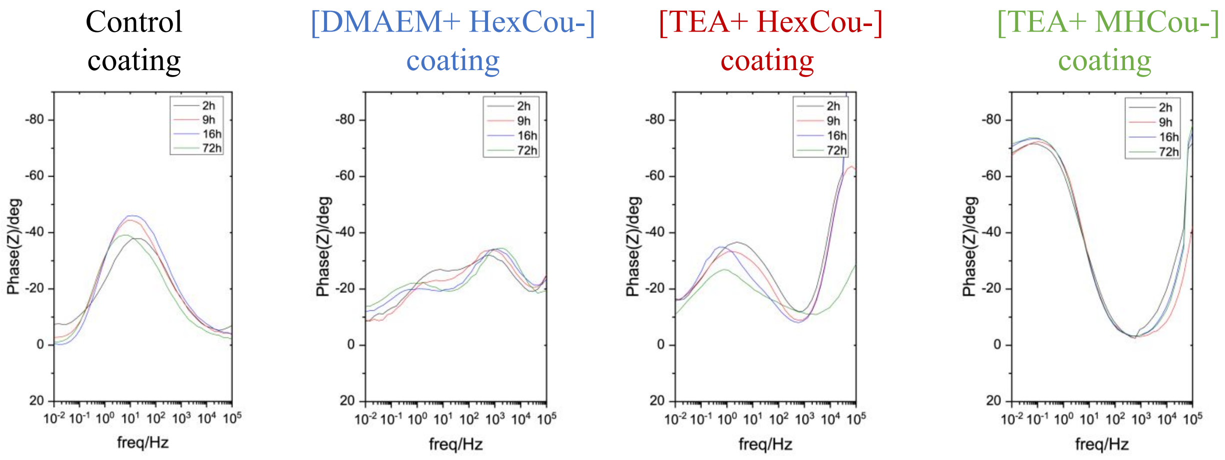

Figure S6. Electrochemical impedance spectra for different polymer coatings on AS1020 mild steel immersed in 0.005M NaCl: phase angle plots for inhibited coating without inhibitors (control) and containing 20% of [DMAEM+ HexCou-], 20% of [TEA+ HexCou-] and 20% of [TEA+ MHCou-] immersed in 0.005M NaCl after 24h
